# Romantic love as regulatory co-option of maternal-bonding circuitry: a testable genetic account

**DOI:** 10.3389/fnbeh.2026.1797790

**Published:** 2026-08-03

**Authors:** Thomas Felesina, Adam Bode

**Affiliations:** 1 School of Psychology, Centre for Psychology and Evolution, The University of Queensland, Brisbane, QLD, Australia; 2 Melbourne School of Psychological Sciences, University of Melbourne, Parkville, VIC, Australia; 3 School of Archaeology and Anthropology, The Australian National University, Canberra, ACT, Australia

**Keywords:** evolution, genetics, mother–infant bonding, regulatory co-option, romantic love

## Abstract

Romantic love lacks a mechanistic, testable evolutionary account. Prior work notes parallels with mother–infant bonding but leaves the genetic architecture and falsifiable predictions unspecified. We argue that romantic love evolved not through the evolution of new “love genes,” but by altering the regulation of existing genes: changing when, where, and under what physiological conditions these genes are active. This regulatory co-option framework generates several falsifiable predictions: (1) a shared genetic basis between maternal bonding and romantic love, (2) the involvement of non-coding, regulatory DNA regions in the brain, (3) a largely shared genetic architecture between the sexes, with differences in gene expression, and (4) genetic signals localized to specific brain circuits. We outline specific genomic and statistical methods to test these predictions, establishing clear criteria that would support or refute our hypothesis. This work shifts the study of romantic love’s evolution from analogy to a program of empirical inquiry and hopes to shed light on how social monogamy entered the *Homo sapiens* lineage. Key challenges include the lack of high quality meta-analytic fMRI evidence of romantic love and the scarcity of detailed data from relevant human brain circuits.

## 1 Introduction

Romantic love remains under-studied despite its centrality to pair-bond formation and family life worldwide (Kowal et al., 2024). Its evolutionary history has received limited attention, with only a small literature addressing functions and origins (Bode et al., 2025a). Some recent work has posited that romantic love may have arisen via co-option of mother–infant bonding in a hominin ancestor of *Homo sapiens* (Bode, 2023) and has begun to outline mechanisms and processes underlying its emergence and maintenance (e.g., Bode and Kavanagh, 2023, 2025; Bode et al., 2025b). Here, we extend that hypothesis by outlining plausible genetic mechanisms of co-option.

### 1.1 Definition of romantic love

We use “romantic love” in the narrow sense throughout: the intense, typically early-stage motivational state directed at a specific individual and characterized by distinctive cognitions, emotions, and behaviors (Bode and Kushnick, 2021), as distinct from the companionate love of long-term established pair bonds. This usage is not idiosyncratic. It follows an established convention in the evolutionary and biopsychological sciences, where the term is applied consistently and aligns with the Collins (2025) English-language and (American Psychological Association, 2018) definitions and with Sternberg’s (1986) triangular framework (Bode, 2026). It is also continuous with foundational relationship science: Berscheid (2010) treats “romantic love,” “passionate love,” “obsessive love,” and being “in love” as alternative names for a single, identifiable phenomenon that recurs across virtually all love taxonomies and is qualitatively distinct from companionate love.

We adopt the following definition:

*a motivational state typically associated with a desire for long-term mating with a particular individual. It occurs across the lifespan and is associated with distinctive cognitive, emotional, behavioral, social, genetic, neural, and endocrine activity in both sexes. Throughout much of the life course, it serves mate choice, courtship, sex, and pair-bonding functions. It is a suite of adaptations and by-products that arose sometime during the recent evolutionary history of humans* (Bode and Kushnick, 2021, p. 21).

Romantic love is most often observed at the start of a romantic relationship, yet it can also arise in the absence of one as unrequited or parasocial love (Bringle et al., 2013; Tukachinsky Foster, 2021). Although variable, it typically lasts around one to two years (see Bode and Kushnick, 2021) and may persist much longer in some individuals (Acevedo and Aron, 2009). Hallmark features include obsessive and idealized thoughts about a loved one, heightened emotional intensity, and strong proximity-seeking or other approach behaviors (Fisher, 1998). Social monogamy is a feature of romantic love, and romantic love may have been the way that social monogamy first entered the *Homo sapiens* lineage (Bode, 2023; Fisher et al., 2016). Because some studies use “romantic love” to refer to any partnered love (e.g., Rinne et al., 2024; Han et al., 2024), we adopt a precise definition in this paper.

Finally, we distinguish the motivational state of romantic love from the behavioral outcome of a pair bond. Although often functionally linked (Bode and Kushnick, 2021), they are not synonymous. Romantic love can occur without a reciprocal partnership (e.g., unrequited love), and stable pair bonds can be maintained by other mechanisms, such as companionate love, after the initial intensity has waned (Bode, 2023). Arranged marriages without romantic love sustain pair bonds through commitment rather than romantic love [the commitment component of Sternberg’s (1986) triangular theory]. Accordingly, our co-option account targets the genetic and neurobiological origins of the motivational state itself, not the evolution of the pair bond. In what follows, we treat romantic love as a proximate mechanism that facilitates the formation of pair bonds, and our genetic framework is concerned with the circuitry underlying this affective–motivational state.

### 1.2 The maternal-bonding co-option account of romantic love

The co-option account of romantic love proposes that the genes implicated in the onset and expression of mother–infant bonding were co-opted in a hominin ancestor of modern humans to support adult bond formation, integrating with existing circuits for courtship-attraction and sexual motivation (Bode, 2023). Comparative work in prairie voles (e.g., Numan and Young, 2016; Walum and Young, 2018) shows overlapping neural substrates for pair bonding and maternal bonding, providing proximate mechanistic support for this possibility. In our use, vole findings identify candidate circuits and regulatory motifs; however, they neither date the putative co-option event nor establish functional homology in hominins. On circumstantial grounds, we tentatively date this co-option to no later than during the existence of *Homo erectus* (∼2 Ma), because marked reductions in sexual dimorphism during this period have been argued to be consistent with social monogamy (Gavrilets, 2012; Plavcan, 2012; Herries et al., 2020). We do note that these are proxy indicators rather than direct evidence. Romantic love may have arisen alongside the evolution of pair bonding; alternatively, it may have predated stable pair bonds, manifesting as a seasonally recurrent or single-breeding-cycle attachment on the lineage leading to *Homo sapiens* (Fisher et al., 2016; Bode, 2023).

Support for the co-option hypothesis is convergent yet indirect (Bode, 2023). Mother–infant bonding and romantic love share psychological signatures—preoccupation (intrusive/idealizing thoughts, doting, caregiving), reciprocity seeking, exclusivity of focus, idealization, heightened responsibility, and proximity seeking—with an early, time-limited period of heightened preoccupation in both states (Leckman and Mayes, 1999). “Baby talk” is also observed in both states (Bombar and Littig, 1996), although it is unclear whether the functions remain the same.

Maternal bonding is not the only conceivable source for romantic love, but we believe it is the strongest candidate on current evidence. Romantic love could in principle have been assembled from any of several pre-existing attachment bonds. We favor maternal bonding on phenotypic grounds: the obsessive, intrusive preoccupation and focused attention of early romantic love closely resemble the early, time-limited preoccupation of mother–infant bonding (Leckman and Mayes, 1999), and are largely absent from friendship, filial bonds, and established companionate relationships. A separate account locates the potential origin elsewhere, proposing that the neural and hormonal machinery of pair bonding was first elaborated for enduring same-sex affiliative bonds in our ape ancestors and only later recruited for reproductive partners (Sandel, 2023, 2025). That account and ours are complementary rather than competing, because they answer different questions: it concerns the origin of the pair bond, whereas ours concerns the origin of romantic love specifically. We therefore present maternal bonding as the most plausible source, while acknowledging that the inference rests largely on resemblance, that the relevant cognitive evidence remains limited, and that alternative or multiple source bonds cannot be excluded.

A plausible temporal cascade is that romantic love arises first, before conception, and helps establish the pair bond (Bode and Kushnick, 2021); during pregnancy, maternal attention begins to reallocate toward the fetus (Leckman and Mayes, 1999); and after birth, mother–infant bonding takes hold (Johnson, 2013), as partner-focused romantic preoccupation recedes. This reciprocal pattern—romantic preoccupation waning as maternal preoccupation rises—is what we would expect if a single bonding system is reallocated from partner to offspring. The two states differ, however, in the timing of their onset: mother–infant bonding is anchored to a fixed reproductive event and begins around birth, whereas romantic love has no comparable trigger and can begin before, during, or after a romantic or sexual relationship (Bode et al., 2025b).

Early neuroimaging points to neural commonalities between mother–infant bonding and romantic love: both engage reward-related subcortical circuits with dense dopamine and oxytocin receptor expression, including the mesolimbic pathway (Bartels and Zeki, 2004). Reviews and meta-analyses further suggest overlap in the ventral tegmental area, amygdala, striatum, and insula; maternal responses often show left-lateralized activation, whereas romantic love tends to be bilateral (Rigo et al., 2019; Shih et al., 2022; Bode and Kushnick, 2021). Both states are associated with measurable increases in circulating oxytocin and endogenous opioids (Ulmer-Yaniv et al., 2016), consistent with current theoretical accounts of the endocrinology of bonding and close relationships (Carter, 2022; Machin and Dunbar, 2011). A key gap is quantitative evidence on the relative intensity of engagement across regions. The only meta-analysis to date directly comparing maternal and romantic love (Shih et al., 2022) found their common activation limited to the ventral tegmental area, but this conjunction did not survive stringent (family-wise error) correction, and its romantic-love inputs were weighted toward long-term, established relationships rather than prototypical romantic love. Fine-grained estimates of regional effect sizes are therefore not yet available. It is also possible that these data have been misinterpreted, and it may be premature to indicate that the left ventral tegmental area is implicated in romantic love (L. Brown, personal communication, 9 July 2026).

### 1.3 Shared neural circuits of maternal bonding and romantic love

**FIGURE 1:**
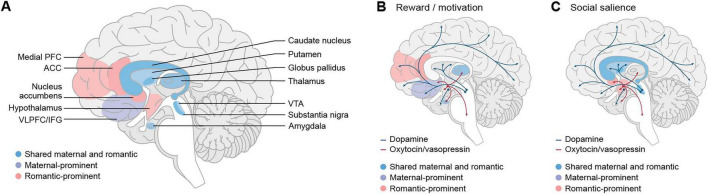
Shared and differentially recruited neural circuits in maternal bonding and romantic love. **(A)** Sagittal view of the regions implicated in maternal bonding and romantic love, color-coded by classification (shared, maternal-prominent, or romantic-prominent). **(B)** The reward and motivation circuit. **(C)** The social salience circuit, each showing the dopaminergic (blue) and oxytocinergic/vasopressinergic (red) projections that modulate these regions.

We organize the neural regions implicated in maternal bonding and romantic love into two functional circuits: a reward and motivation circuit and a social salience circuit (Figure 1). We focus on these two because the human evidence for them is strongest. Other circuits sometimes proposed for bonding (e.g., caregiving and regulatory-control circuits) rest mainly on rodent work and are not clearly defined in human neuroimaging of either state, so we leave them aside. Within this scheme, each region is labeled as shared between the two states, more prominent in maternal bonding, or more prominent in romantic love; these labels are relative, marking where a region stands out across the available studies rather than implying exclusive involvement in either state.

The reward and motivation circuit is the most consistently implicated neural substrate shared by the two states. As delineated in primate anatomy and human imaging, it is organized around the dopaminergic midbrain (i.e., the ventral tegmental area and substantia nigra) together with their dorsal and ventral striatal targets, including the caudate, putamen, and globus pallidus (Haber and Knutson, 2010). The ventral tegmental area and substantia nigra are the principal origins of the ascending dopaminergic projections that innervate the striatum and forebrain (Sesack and Grace, 2010; Wang et al., 2020; Xu and Yang, 2022), providing a plausible substrate through which a common neuromodulatory system could be engaged by either an infant or an adult partner. The maternal meta-analysis of Rigo et al. (2019) provides the strongest evidence for involvement of these midbrain–striatal nodes, and romantic-love reviews report the same regions (Acevedo et al., 2025; Bode and Kushnick, 2021), supporting their classification as shared; the more recent meta-analysis of parent neural responses (Niehaus et al., 2026) emphasizes broader frontoinsular, corticolimbic, temporal, and sensory convergence rather than reward-circuit recruitment specifically. However, there may be inconsistencies between the coordinates reported for brain activity and the structures stated to be implicated. In particular, the figures accompanying Rigo et al. (2019) appear to show that the activation common across the studies falls in the hypothalamus rather than in the midbrain substantia nigra/ventral tegmental area as labeled (L. Brown, personal communication, 9 July 2026). We therefore treat these regional assignments as provisional. The nucleus accumbens and hypothalamus appear in both literatures but are reported more consistently in romantic-love studies than in the maternal meta-analytic data—the accumbens being subsumed within broader dorsal-striatal findings in mothers (Rigo et al., 2019), and the hypothalamus appearing in romantic-love imaging (Acevedo et al., 2025; Bode and Kushnick, 2021) but not in the maternal meta-analysis—and are therefore provisionally classified as romantic-prominent.

The social salience circuit is anchored cortically in the anterior insula and dorsal anterior cingulate cortex and extends to limbic and subcortical nodes, including the amygdala, thalamus, hypothalamus, and brainstem structures (Seeley et al., 2007; Menon and Uddin, 2010; Seeley, 2019). Across both maternal and romantic states, the amygdala (Rigo et al., 2019; Acevedo et al., 2025; Bode and Kushnick, 2021), anterior insula (Rigo et al., 2019; Niehaus et al., 2026; Acevedo et al., 2025; Bode and Kushnick, 2021), and thalamus (Rigo et al., 2019; Niehaus et al., 2026; Acevedo et al., 2025) recur, alongside regions showing state-biased recruitment. The ventrolateral prefrontal cortex and inferior frontal gyrus feature more prominently in the maternal literature (Rigo et al., 2019; Niehaus et al., 2026). By contrast, the medial prefrontal cortex and anterior cingulate cortex are reported more consistently for romantic love (Acevedo et al., 2025; Bode and Kushnick, 2021). Whereas the reward circuit is principally dopaminergic, the social salience circuit is strongly modulated by the neuropeptides oxytocin and vasopressin, whose receptors are distributed across the amygdala, insula, cingulate, hypothalamus, and striatum in the human brain and which bias the assignment of salience to socially significant stimuli (Meyer-Lindenberg et al., 2011; Acevedo et al., 2025). This neuropeptidergic modulation provides a direct point of contact with the regulatory co-option account developed below, because the receptor systems whose expression we hypothesize to be retuned by cis-regulatory change (e.g., OXTR, AVPR1A) are concentrated in precisely these circuits.

We stress an asymmetry in evidential weight across these classifications. The maternal assignments rest on quantitative meta-analyses (Rigo et al., 2019; Niehaus et al., 2026), whereas the romantic-love assignments derive largely from narrative reviews and a single, underpowered meta-analysis (Shih et al., 2022; Acevedo et al., 2025; Bode and Kushnick, 2021). A well-powered meta-analysis of romantic-love fMRI studies is therefore required before the shared status of these nodes can be regarded as established. For the purposes of Figure 1 we rely on Rigo et al. (2019) and Acevedo et al. (2025), and the classifications shown there should be read as a provisional summary of those sources rather than as settled anatomy. The romantic-love side of that summary is further limited by what its constituent studies measured: the review draws on work indexing long-term intense romantic love (Acevedo et al., 2012) and on earlier, anatomically less precise imaging (e.g., Bartels and Zeki, 2004), neither of which should properly count toward prototypical, early-stage romantic love as we define it here. Research establishing the actual neural overlap between maternal bonding and romantic love, and the regions specific to each, will require a more precise meta-analysis of both states.

## 2 Co-option as an evolutionary process

Co-option refers to the evolutionary redeployment of an existing biological component to a new function. Although co-option can also proceed through protein-coding change, we adopt a deliberately narrowed sense here: redeployment via cis-regulatory change, so the component performs a new function without altering the encoded protein. Co-option in this sense is the mechanistic counterpart of exaptation (Gould and Vrba, 1982) (i.e., the recruitment of an existing feature to a new function) and we use “co-option” to denote specifically the regulatory route to such exaptation. Across taxa, much phenotypic novelty arises by reusing a conserved toolkit of pleiotropic genes through regulatory evolution rather than by inventing new genes (e.g., Wittkopp and Kalay, 2012; Xu et al., 2009). In eukaryotes, cis-regulatory elements (enhancers, promoters, silencers, insulators) control when, where, and how strongly a gene is transcribed. They are evolutionarily labile, especially enhancers, with transcription-factor binding sites gained and lost between lineages and whole elements gaining, losing, or shifting activity over evolutionary time (Schmidt et al., 2010; Villar et al., 2015; Wray, 2007). Co-option can occur when non-coding variants alter enhancer–promoter control of a shared locus. For example, single-nucleotide substitutions that create or strengthen transcription-factor motifs, repeat-length changes in regulatory microsatellites, transposable-element insertions that donate binding sites, or variants that change 3D enhancer–promoter looping. To a first approximation, mutations in one enhancer shift expression mainly in the module it controls, leaving other enhancer-driven patterns largely intact, though this modularity is partial: enhancers can be pleiotropic and redundant (e.g., shadow enhancers), so functional independence is a matter of degree (McDonald and Reed, 2024). While promoter changes can matter, cis-regulatory divergence is generally more attributable to enhancer alterations than promoter alterations (Brown and Feder, 2005; Wray, 2007). Enhancers contain clustered binding sites for multiple transcription factors: proteins that switch target genes on or off and set where and how strongly each is transcribed. Through these sites, enhancers integrate lineage/identity inputs with state-dependent inputs (Ong and Corces, 2011). A single gene commonly has several enhancers, each tuned to a particular combination of cell type, developmental stage, or physiological state. In practice, selection could increase expression of a “love-relevant” gene in adult partner-processing circuits, without disturbing expression in postpartum caregiving circuits that are governed by different enhancers.

## 3 Co-option of mother–infant bonding to romantic love

**FIGURE 2:**
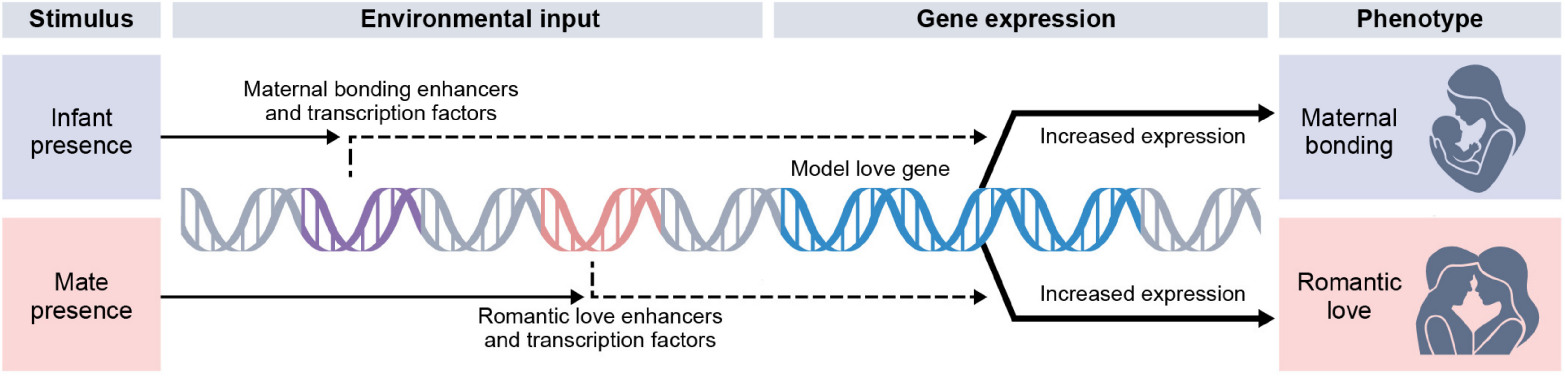
Regulatory co-option model for romantic love. Environmental cues (partner vs. infant) shift endocrine/neuromodulatory state and transcription-factor availability, producing context-specific binding at enhancer modules (“romantic-love” vs. “maternal-bonding”). The same effector genes are thereby expressed in different circuits, at different times and levels, yielding partner-directed romantic love or infant-directed caregiving, without any change to protein sequence. One locus is shown for clarity; the architecture is expected to be highly polygenic and largely regulatory.

Applied to romantic love, we propose that selection co-opted regulatory control of genes already engaged in mother–infant bonding, so that their expression increased in adult partner-processing circuits (see Figure 2). The proteins remain unchanged; what shifts are (i) spatial expression (which cells/brain regions), (ii) temporal expression (when expression turns on), and (iii) quantitative output (how much transcript/protein is produced). Different combinations of transcription factors on distinct enhancers can therefore redeploy the same gene to different bonding contexts without cross-talk (i.e., the maternal and partner-directed deployments do not interfere). Whatever the specific transcription factor constellation enabling the hypothesized co-option, the predicted net effect is higher receptor or peptide dosage (e.g., oxytocin, vasopressin) in specific adult circuits while leaving postpartum modules intact. Hormone-responsive motifs (estrogen/androgen/glucocorticoid receptors) acting with other trans-acting regulators whose levels/activity vary with sex, life stage, or physiological state make enhancers operate as context-sensitive switches that turn a gene on only in the relevant sex or at the right time (Wray, 2007). Such sex- or stage-limited expression reduces intralocus sexual conflict—the problem that the same allele can benefit one sex but harm the other. Empirically, variants that tweak these switches usually appear as cis-expression quantitative trait loci (cis-eQTLs)—alleles associated with higher or lower expression of a nearby gene—or as allele-specific expression (one allele expressed more than the other) (Wittkopp and Kalay, 2012; Wray, 2007). As shown in Figure 2, cues from the environment (such as whether a partner or infant is present) shift hormone and neuromodulator levels in the brain, which in turn change which transcription factors are available and where they bind in the genome. This state-dependent binding at enhancer modules turns the same downstream genes on in different neural circuits, at different times and intensities, effectively “retuning” expression without altering the underlying protein-coding sequence.

## 4 Quantitative-genetic consequences and empirical status

If the same loci influence both maternal bonding and romantic love, then selection acting in one context (initially maternal care) can yield a correlated response in the other (adult attachment). Under this scenario, the relevant genes are most plausibly autosomal (present in both sexes), so alleles are exposed to selection in females yet remain present in males, generating positive between-sex genetic correlation and correlated evolution (Felesina, 2025). Sex-linked architectures are not excluded in principle, but Y-linkage is impossible for female selection, and X-linkage introduces dosage and inheritance asymmetries that complicate the simple “selected in mothers, expressed in both” logic (Deng et al., 2014). Candidate “bonding genes” most often invoked in this literature (i.e., OXTR [3p25.3], AVPR1A [12q14.2], OPRM1 [6q24–q25], CD38 [4p15], DRD2/DRD4 [11q/11p], COMT [22q11.2]) meet this criterion: all are autosomal (Acevedo et al., 2020; Bakermans-Kranenburg and van IJzendoorn, 2008; Chen et al., 2011; Khani et al., 2023; Makhanova et al., 2021; Tchalova et al., 2021; Walum et al., 2008). These loci are cited as illustrative within a highly polygenic architecture; we do not treat candidate-gene associations as decisive. Consistent with cis-regulatory tuning, variation in the AVPR1A RS3 microsatellite—located in the 5’ regulatory region—relates to human pair-bonding and caregiving phenotypes. In men, the 334-bp RS3 allele associates with lower partner-bonding and marital outcomes (Walum et al., 2008); in fathers, 334-carriers show attenuated left inferior anterior prefrontal cortex (APFC) responses to their own child (Nishitani et al., 2017); and in mothers, long (≥334 bp) RS3 indirectly predicts lower observed maternal sensitivity via cry-processing cognitions (Leerkes et al., 2017). Such regulatory variation supports the idea that modulation of vasopressin receptor expression (rather than protein sequence changes) helped create diversity in bonding behavior and could be a substrate for co-option. A 5’-flanking microsatellite in vole *Avpr1a* (Lim et al., 2004) is hypothesized to tune region-specific expression and social behavior. Barrett et al. (2013) discuss analogous evidence for human/chimpanzee AVPR1A RS3 (e.g., associations with men’s relationship quality) and, using shRNA, show that reducing V1aR in ventral pallidum causally attenuates partner-preference formation in prairie voles. Furthermore, it has recently been shown in prairie voles that motherhood re-tunes expression of the same “bonding genes” across pair-bond and maternal circuits. In pair-bonded females, oxtr, d2r, and opioid receptor transcripts rise in nucleus accumbens, and oxtr, d1r/d2r, and opioid receptors rise in medial preoptic area; anterior cingulate expression scales with bond duration. Coordination across nodes also shifts: ACC–NAc co-expression for oxtr and d1r appears only in pregnant/postpartum females. These state- and circuit-specific dosage changes align with a cis-regulatory co-option model in which context-limited regulatory inputs (e.g., hormone-responsive transcription factors) adjust expression without altering protein sequence (Forero et al., 2024).

Vole work has been informative about bond *formation*, where dopamine–oxytocin interactions in the nucleus accumbens—acting on D1- and D2-type medium spiny neurons—gate the transition from mating to selective partner preference (Walum and Young, 2018; Loth and Donaldson, 2021). Three caveats temper its evidential weight, however. First, prairie voles are socially monogamous rodents whose evolutionary history is distant from that of hominins, so shared mechanisms cannot be assumed to be homologous. Second, much vole research concerns bond maintenance and the consequences of partner loss rather than formation, and has relied heavily on virgin animals forming a first pair bond—a paradigm of limited relevance to human social behavior (e.g., Kenkel et al., 2019). Third, the causal centrality of specific signals has recently been questioned: oxytocin-receptor-null voles still form pair bonds and display relatively normal parental care (Berendzen et al., 2023). We therefore treat vole findings as a source of candidate mechanisms and regulatory motifs, not as direct evidence for the human case.

Although we highlight particular genes, such as those related to oxytocin and vasopressin as illustrative, we expect a highly polygenic, cis-regulatory architecture in which many loci of small effect contribute to bonding phenotypes, as is the case with most psychological and behavioral traits with graded variation across a population (Plomin, 2008). Under this view, co-option reflects coordinated shifts across multiple enhancers rather than dependence on any single “master” gene (Wray, 2007; Wittkopp and Kalay, 2012). The behavioral pleiotropy we posit—that the same regulatory variants contribute to both maternal bonding and pair bonding in humans—remains inferential. Comparative evidence and human observations on maternal bonding and romantic love make the hypothesis plausible, but cell-type–resolved, region-specific cis-eQTL evidence and causal perturbations in the implicated circuits are still lacking. We therefore frame it as an empirically grounded expectation, not a settled fact.

## 5 From regulatory variant to bonding behavior

It is worth making the hypothesized causal chain explicit, since our argument spans four levels: regulatory sequence, gene expression, circuit signaling, and behavior. At the molecular level, the cis-regulatory variants discussed above (e.g., AVPR1A RS3, or putative OXTR enhancer variants) would be expected to alter transcription-factor binding at enhancer modules and thereby shift the density of vasopressin and oxytocin receptors (V1aR and OXTR) within particular circuits rather than across the brain (Wray, 2007). Where receptor availability is raised in the social-salience circuit (i.e., amygdala, anterior insula, hypothalamus), the partner should, in principle, be assigned greater salience as a social stimulus (Walum and Young, 2018; Meyer-Lindenberg et al., 2011; Shamay-Tsoory and Abu-Akel, 2016). In parallel, altered dopamine-receptor dosage (e.g., DRD2/DRD4) in the mesolimbic reward circuit (VTA → nucleus accumbens) would plausibly amplify the reward evoked by partner proximity and strengthen approach motivation. Because oxytocinergic salience and dopaminergic reward signaling appear to be coupled at these nodes (Liu and Wang, 2003; Loth and Donaldson, 2021; Scheele et al., 2013), we tentatively suggest that it is coordinated retuning across them, rather than change at any single locus, that would orient the system toward one highly salient and rewarding target. At the behavioral level, such changes would be expected to surface as the hallmark features noted earlier (i.e., proximity-seeking, partner-focused preoccupation, idealization, and exclusivity) redirected onto an adult partner rather than an infant (cf. Leckman and Mayes, 1999). We emphasize that this chain is, at present, mechanistically plausible rather than demonstrated: each link is inferred from animal models and human correlational data, and direct human evidence remains lacking.

## 6 Predictions

If romantic love co-opts circuitry used for mother–infant bonding, the two phenotypes should share polygenic signal: specifically, a positive cross-trait genetic correlation (*r*_*g*_ > 0) and evidence that at least some associated regions harbor the same causal variant in both traits. This follows because co-option implies partial overlap of causal variants, not entirely novel architecture. However, *r*_*g*_ measures genome-wide alignment of SNP effects and cannot distinguish a single common source from multiple pathways. This can be tested by: (i) estimating *r_g_*from GWAS using LDSC or bivariate GREML; (ii) modeling a shared latent factor with Genomic SEM; and (iii) using fine-mapping plus cross-trait colocalization to identify shared causal loci.

Because the genetic overlap is hypothesized to be largely regulatory, not coding, effects should be enriched in non-coding, cis-regulatory variants that are state-, region- and cell-type–specific, rather than in protein-altering changes. Co-option by regulatory “retuning” is the most parsimonious route to redeploy existing bonding circuits for pair-bonding. This could be tested with stratified/partitioned heritability (S-LDSC) using brain enhancer/promoter annotations (bulk and single-cell/ATAC), TWAS/eQTL integration (e.g., PsychENCODE/GTEx; GTEx Consortium, 2020) to show cis-regulatory mediation, and burden/enrichment contrasts showing under-representation of protein-coding variants. If the phenotype indexes romantic love (rather than infant caregiving), for example, as in Bode and Kavanagh (2025), cis-regulatory enrichment should preferentially localize to brain regions implicated in romantic love: the ventral tegmental area, nucleus accumbens, amygdala, medial prefrontal cortex, anterior cingulate cortex, insula, caudate, and putamen (Bode and Kushnick, 2021). Conversely, caregiving-specific phenotypes should preferentially enrich in the ventral tegmental area, substantia nigra, ventrolateral prefrontal cortex, insula, amygdala, and striatum (Rigo et al., 2019). This can be tested by cell-type/tissue-specific enrichment (S-LDSC with chromatin maps and cell-type expression profiles), spatial-transcriptomic overlap with these regions, and TWAS/eQTL signals anchored to those circuits.

Finally, the framework makes a specific prediction about the sex differences observed in romantic love. Sex differences in its expression have been reported (Bode et al., 2025b): in a WEIRD sample of young adults, men reported falling in love slightly more often and roughly a month earlier than women, whereas women reported somewhat greater intensity and more frequent partner-focused obsessive thoughts (Bode et al., 2025b). Commitment differed significantly between the sexes under univariate but not multivariate analysis, and some effects replicated prior work (e.g., Galperin and Haselton, 2010; Watkins et al., 2022) while others appeared novel. Such patterns are often interpreted within a sexual-selection framework, in which earlier male onset may function as an honest signal of commitment to choosier females and may facilitate courtship (Bode et al., 2025b); notably, however, an early fMRI study found no significant sex differences in neural activity when participants viewed images of a loved one (Aron et al., 2005). Any phenotypic divergence does not necessarily imply sex-unique loci. If shared circuitry is read through sex-differential hormonal milieus rather than sex-unique genes, we expect high cross-sex genetic correlation for broad bonding liability, with sex-differential expression (not distinct loci, though X-linked loci may also contribute to resolving sexual conflict) accounting for phenotypic divergence. This can be tested with sex-stratified GWAS to estimate *r*_*g*_ (male, female) and with sex-interaction eQTL/TWAS and steroid-responsive enhancer annotations (puberty/early adulthood vs. late pregnancy/early postpartum) to demonstrate regulatory, hormone-linked readouts.

Because large genomic studies are costly, we outline several low-cost tests that do not require new genotyping. These designs cannot identify specific circuits or demonstrate genetic causality, but they can show whether patterns in existing data are at least consistent with the idea that maternal bonding mechanisms have been redeployed for romantic love. First, family studies offer a low-cost method to probe whether romantic love sits on top of a broader, heritable bonding liability. While regulatory evolution allows for some decoupling of traits within an individual, the hypothesis predicts that the underlying genetic sensitivity (the shared bonding factor) should aggregate in families. Researchers could recruit nuclear families or sibling sets to assess whether romantic-love phenotypes (e.g., intensity, intrusive thoughts, approach behaviors) and caregiving propensities (e.g., tenderness toward infants, willingness to sacrifice) co-segregate. Using multilevel modeling, one can test whether families that are, on average, higher in caregiving liability also tend to be higher in romantic-love intensity. This design allows researchers to test for “discordant pleiotropy,” a phenomenon where the same heritable sensitivity manifests as different behaviors depending on the individual’s context. For instance, a shared high-bonding variant might manifest as intense romantic preoccupation in a young man, yet appear as heightened maternal responsiveness in his postpartum sister.

Within this general design, cross-sex resemblance is informative regarding the shared genetic architecture between men and women. By comparing correlations in sister–sister, brother–brother, and brother–sister pairs, one can test whether male and female romantic phenotypes tap into the same familial factors. Under the autosomal inheritance we posit, where genes are shared but regulation is sex-differential, we expect modest but positive similarity even in opposite-sex pairs, with same-sex pairs likely showing higher concordance due to shared hormonal milieus. In contrast, near-zero brother–sister resemblance, alongside strong same-sex resemblance, would point toward distinct, sex-specific genetic architectures, thereby weakening the claim that both sexes rely on a common, co-opted bonding system.

Second, our model suggests that transitions between romantic love and bonding states may trigger a reallocation of bonding resources from one target to the other. Longitudinal studies tracking women from early pregnancy into the postpartum period could test this prediction. Such studies must recruit participants who are still in the intense, early phase of romantic love (typically less than two years) to capture potential trade-offs. If the same underlying genetic architecture is indeed being tuned by different regulatory mechanisms, the onset of intense maternal preoccupation may necessitate a temporary reduction in romantic preoccupation, distinct from the effects of fatigue that are likely associated with early maternal care. Observing such an inverse relationship, where the rise of one bonding state predicts the decline of the other, would support the view that these two drives regulate access to the same shared neurobiological resources. Conversely, the formation of a new, intense romantic bond during a period of offspring dependence offers a test of the inverse trade-off. Our regulatory co-option framework implies that the upregulation of courtship- and attraction-related signaling may competitively inhibit the neural machinery required for high-investment caregiving. These predictions arising from the co-option hypothesis may offer a specific proximate mechanism for aspects of the “Cinderella” effect (Daly and Wilson, 1988): the observation that stepchildren face significantly higher risks of abuse and neglect than children living with two biological parents. If romantic and maternal states rely on the same cis-regulatory switches, the physiological intensity of a new romance could functionally suppress parental vigilance.

The genetic predictions above can be complemented at the neural level. As elsewhere in the neural account, the maternal side rests on stronger meta-analytic evidence than the romantic-love imaging literature, so these are framed to be falsifiable rather than confirmatory. First, a well-powered meta-analysis of romantic-love fMRI studies, drawing on the corpus catalogued by Bode and Kowal (2023), would be expected to recover the reward and motivation regions established for maternal bonding by meta-analysis (the ventral tegmental area and substantia nigra, caudate, putamen, and globus pallidus; Rigo et al., 2019, though see the caveat above regarding the correspondence between reported coordinates and named structures). Whether the ventral tegmental area in particular emerges as a shared node remains open: the only existing comparison reports a VTA conjunction that did not survive stringent correction and whose interpretation is in any case uncertain (Shih et al., 2022), so it is premature to treat the region as established for romantic love. Adequately powered data are needed to determine whether the overlap is real, and failure to recover it would weaken the shared-substrate claim.

Shared activation is necessary for the account but does not by itself favor it over the alternatives, since any two rewarding attachment states should recruit common reward circuitry. A more diagnostic prediction concerns the specificity of the resemblance: if romantic love was built on maternal-bonding circuitry in particular, its neural signature should resemble that of maternal (mother–infant) bonding more closely than that of other strong bonds such as close friendship — the neural counterpart of the proposal that romantic love’s preoccupying profile resembles mother–infant bonding rather than friendship. Multivariate or representational-similarity analyses across love types (e.g., Rinne et al., 2024) finding romantic love no closer to parental love than to friendship would favor a non-maternal origin (cf. Sandel, 2023) or general convergence. Two limits apply: this tests which bond was the source, not whether the mechanism was regulatory co-option (addressed by the genetic predictions above); and because closer bonds recruit reward circuitry more strongly in general (Rinne et al., 2024), romantic–maternal similarity must be judged against other equally close bonds, so the test reflects shared organization rather than shared intensity.

## 7 Discussion

Building on Bode (2023)’s co-option hypothesis that romantic love may have, in part, emerged from biological circuitry involved in mother–infant bonding, we outline plausible genetic and evolutionary processes supporting this account, linking proximate mechanisms to Bode (2023)’s ultimate explanation. Our aim is to make the hypothesis testable, not to declare it established. We posit that existing bonding circuitry was reused via changes in when, where, and under which physiological states the same genes are expressed, rather than via the evolution of entirely new “love genes.” In other words, regulatory retuning—not new protein-coding sequence—could map shared genetic inputs onto romantic love phenotypes.

This proposal is genetically plausible on three grounds. First, social-attachment phenotypes in mammals are likely highly polygenic and, in large part, autosomal, so alleles that influence maternal bonding are present in both sexes and can, in principle, be expressed in adult pair-bond circuits. Second, adaptive novelty in complex traits commonly arises through cis-regulatory changes—altered enhancer–promoter control that is specific to cell type, developmental period, or physiological state—allowing expression to increase in circuits implicated in adult romantic love without disrupting postpartum caregiving modules. Third, sex- and state-limited regulation (e.g., hormone-responsive enhancers) can translate the same variants into different outcomes across contexts, reducing pleiotropic costs while enabling redeployment. Together, these points are consistent with basic quantitative-genetic expectations: if romantic love co-opts maternal-bonding pathways, we should observe positive genetic covariance between traits indexing these two domains. Furthermore, this proposal is consistent with recent large-scale comparative work that identified a conserved transcriptomic signature associated with the repeated, independent evolution of social monogamy across vertebrates (Young et al., 2019). The robustness of this conserved signal has been contested (Jiang and Zhang, 2019; see Young and Hofmann, 2019, for a reply), so we treat it as suggestive rather than definitive. While Young et al. (2019) examined the behavioral suite of monogamy rather than the specific affective state of romantic love, their findings provide proof-of-principle that complex social behaviors can arise from the convergent redeployment of homologous gene networks.

Beyond the predictions outlined above, comparative work across pair-bonding species may reveal parallel regulatory changes at orthologous loci (e.g., OXTR/AVPR1A regulatory regions), offering a mechanistic proof-of-principle for the convergent redeployment of social bonding circuitry, even if the specific phenotype of romantic love is not shared. Seasonally monogamous species [e.g., many fox (*Vulpes*) species; MacDonald et al., 2019], which form intense but time-limited bonds tied to a breeding cycle, could provide a valuable complementary model.

A general caveat applies across the evidence reviewed here. The mechanistic, causal evidence for bonding circuitry derives almost entirely from animal models, whereas the human evidence is correlational or indirect (i.e., neuroimaging associations, endocrine correlations, and candidate-gene findings). These studies are not experimental. This asymmetry raises the question of homology versus analogy: shared activations or shared neuromodulators need not imply that the same circuit performs the same function across species. The concern is concrete rather than abstract, because oxytocin and vasopressin receptor distributions differ markedly between rodents and primates, and translation from one to the other is not straightforward (Freeman and Young, 2016). Notably, chimpanzees and most other anthropoid primates largely lack the striatal oxytocin-receptor expression (e.g., in the nucleus accumbens) that is central to the rodent pair-bonding model, whereas humans appear to possess it (Rogers Flattery et al., 2022). We therefore treat cross-species correspondences as analogies to be tested rather than as established homologies.

There are important limitations. In humans, most mechanistic evidence is indirect; cell-type–specific regulatory data from relevant brain circuits (single-cell eQTL, chromatin accessibility, enhancer activity) are still sparse. Phenotyping of “romantic love” is heterogeneous in onset, intensity, and time course, and measurement error will attenuate genetic signals and complicate cross-trait analyses with maternal bonding measures. A further psychometric challenge is ensuring that measures of intense, early-stage romantic love are not confounded with related but theoretically distinct constructs, such as sexual desire or long-term companionate attachment (Fisher, 1998), which may have partially dissociable biological underpinnings. Disentangling these phenotypes in large-scale genomic studies will be critical for isolating the specific genetic architecture of romantic love. Prior candidate-gene findings—often centered on OXTR/AVPR1A—are illustrative (Border et al., 2019; Duncan and Keller, 2011) but not definitive; small studies rarely produce robust signals in complex traits, so genome-wide, preregistered designs are needed. Co-option from maternal bonding is plausible but not exclusive; other evolutionary processes are likely to have been involved, such as sexual selection for reliable signals of partner commitment and parental investment (Bode et al., 2025b). Finally, if shared variants influence caregiving and adult attachment, correlated responses could impose costs in some contexts (e.g., maladaptive preoccupation, attachment anxiety), and gene-by-environment interactions—via hormones, early caregiving, or social context—likely gate expression in ways that current cross-sectional designs cannot resolve. The cultural environment is a particularly salient component of this social context; cross-cultural variation in mating systems, social norms, and display rules may interact with genetic predispositions to shape the ultimate expression and behavioral consequences of romantic love.

Beyond these general considerations, several limitations are specific to human research. First, the neural evidence base is itself underpowered: a quantitative fMRI meta-analysis exists for the maternal response (Rigo et al., 2019), but no comparably well-powered meta-analysis is yet available for romantic love specifically, so the regional effect sizes used to anchor our circuit-level predictions remain provisional (Shih et al., 2022). Two further problems compound this. The coordinates reported in the source literature and the structures named as implicated do not always agree (L. Brown, personal communication, 9 July 2026), and the single existing cross-state comparison may have misinterpreted its data, so it is premature to treat the left ventral tegmental area as implicated in romantic love (L. Brown, personal communication, 9 July 2026). The circuit assignments in Figure 1 accordingly rest on Rigo et al. (2019) and Acevedo et al. (2025) as the best available sources; a more precise meta-analysis of both states will be needed before the overlap we describe can be treated as established anatomy. Second, and compounding the cross-cultural considerations noted above, the human literature draws disproportionately on WEIRD (Western, educated, industrialized, rich, democratic) samples (Bode and Kowal, 2023), limiting how far any estimated genetic architecture can be generalized across populations. Third, no GWAS with an adequate romantic-love phenotype currently exists. Fourth, because participants are typically recruited from within established relationships, the early-stage motivational state is difficult to isolate from ongoing pair-bond maintenance, and the two are readily conflated in cross-sectional human samples. This applies with particular force to the human genetic evidence: the one informative genetic study (Acevedo et al., 2020) indexed romantic-love maintenance and gene-by-state interactions rather than prototypical romantic love. Finally, the causal manipulations that license strong inference in vole models are neither ethical nor practical in humans; human evidence will therefore remain largely correlational, and so the extrapolation of mechanistic homology from rodents to hominins is provisional.

In sum, we offer a testable account of Bode’s (2023) hypothesis. We refine earlier versions that invoked gene duplication: instead of new copies that neofunctionalized, we argue the same pleiotropic loci were repurposed by cis-regulatory changes that alter when, where, and in which physiological states they are expressed. Human studies should develop reliable, state-aware measures of romantic-love intensity and trajectory, conduct well-powered GWAS, estimate SNP-heritability and autosome versus X contributions, and test cross-trait genetic correlations with maternal-bonding phenotypes. Functional genomics should assess colocalization of trait loci with brain cis-eQTL and enhancer activity with attention to sex- and state-limited effects. If romantic love reflects regulatory co-option of maternal-bonding programs, then (i) maternal-bonding and pair-bond phenotypes should exhibit positive genome-wide genetic correlation and load on a shared latent liability in Genomic SEM; (ii) SNP effects should be enriched in non-coding, cis-regulatory annotations that are state-, region-, and cell-type–specific (with relative depletion of protein-altering variation); (iii) a subset of signals should colocalize across eQTL/TWAS in candidate circuits (e.g., the midbrain, amygdala, striatum, ventrolateral prefrontal cortex, and insula in maternal bonding; Rigo et al., 2019; the ventral tegmental area, nucleus accumbens, amygdala, medial prefrontal cortex, anterior cingulate cortex, insula, caudate, putamen in romantic love; Bode and Kushnick, 2021), with Q_SNP distinguishing shared from trait-specific effects; and (iv) architecture should be largely autosomal and sex-shared, with context-dependent expression. These predictions are adjudicable with existing resources (large-sample GWAS, baseline-LD S-LDSC using brain single-cell/ATAC annotations, PsychENCODE/GTEx TWAS/colocalization) under a preregistered analysis plan. Disconfirmation (e.g., absence of a common factor, coding-variant enrichment, or mismatched tissue/cell-type profiles) would count against the co-option account; either way, moving from analogy to quantitative tests advances the field.

## Data Availability

The original contributions presented in the study are included in the article/supplementary material, further inquiries can be directed to the corresponding author/s.
